# Clinicopathological features of combined hepatocellular-cholangiocarcinoma with sarcomatous change: Case report and literature review

**DOI:** 10.1097/MD.0000000000009640

**Published:** 2018-01-19

**Authors:** Qianru Gu, Xia Yu, Hanbin Chen, Guorong Chen

**Affiliations:** Department of pathology, First Affiliated Hospital of Wenzhou Medical University, Ouhai, Wenzhou, People's Republic of China.

**Keywords:** cholangiocarcinoma, hepatocellular carcinoma, sarcomatous changes

## Abstract

**Rationale:**

Combined hepatocellular-cholangiocarcinoma (cHCC-CC) is a rare subtype of primary liver malignancy comprising <1.5% of all primary liver tumors. Sarcomatoid changes in cHCC-CC are even rarer. Due to the rarity of this subtype, its clinicopathological feature is poorly understood. Therefore, here we report 2 tumors.

**Patient concerns:**

The first patient was a 44-year-old man with 5-year history of hepatitis B-induced cirrhosis. The resection of right liver revealed a 2.5 × 2.5 × 2 cm tumor mass. Histologically, the tumor showed areas of the typical moderately differentiated HCC. An intermingled adenocarcinoma with pleomorphic and spindle-shaped cells was also identified. The second case involved a 54-year-old man with a history of hepatitis B-induced cirrhosis. A 3.5 × 3 × 3 cm mass was found in the middle left of falciform ligament. Microscopically, the tumor consisted of spindle-shaped sarcomatoid carcinoma cells mixed with typical well-differentiated HCC and well-differentiated CC.

**Diagnoses:**

According to the clinicopathological features, diagnosis of cHCC-CC with sarcomatous change was made.

**Interventions:**

In the first case, right lobectomy of the liver was performed. The second patient underwent laparoscopic, hepatic left lateral lobectomy.

**Outcomes:**

The first patient was alive and well 10 years after the surgical resection without additional treatment. In second case, at 8 months after surgical resection, there was no evidence of recurrence or metastasis.

**Lessons:**

In this report, we describe 2 rare cases of cHCC-CC with sarcomatous change, and findings are helpful for the pathologists would like to further identify the clinicopathological features of this rare tumor.

## 1 Introduction

Combined hepatocellular-cholangiocarcinoma (cHCC-CC) was a rare subtype of primary liver cancer, especially when it had coexisting of sarcomatous component. To our knowledge, only 8 cases had been reported in the literature.^[1–8]^ Here we described additional 2 cases with immunohistochemical characteristics. Both patients gave consent for these studies and their publication.

## 2 Case presentation

### 2.1 Case 1

An asymptomatic, 44-year-old man, who had 5-year history of hepatitis B-related cirrhosis, underwent a routine ultrasound examination of liver that demonstrated an isolated mass in the right lobe of the liver. Complete blood cell count on admission revealed a decreased platelet count of 76 × 10^9^/L (100–300 × 10^9^/L). His liver function tests were normal. His serum alpha-fetoprotein (AFP) level was 20.1 μg/L (0–20 μg/L). Serology was positive for viral hepatitis B and negative for viral hepatitis C carrier status. Right lobectomy of the liver was performed. On postoperative day 40, his serum AFP level had decreased to normal (3.9 μg/L). The patient's postoperative course was uneventful and he had already lived for 10 years after surgery with no additional treatment.

**Figure 1:**
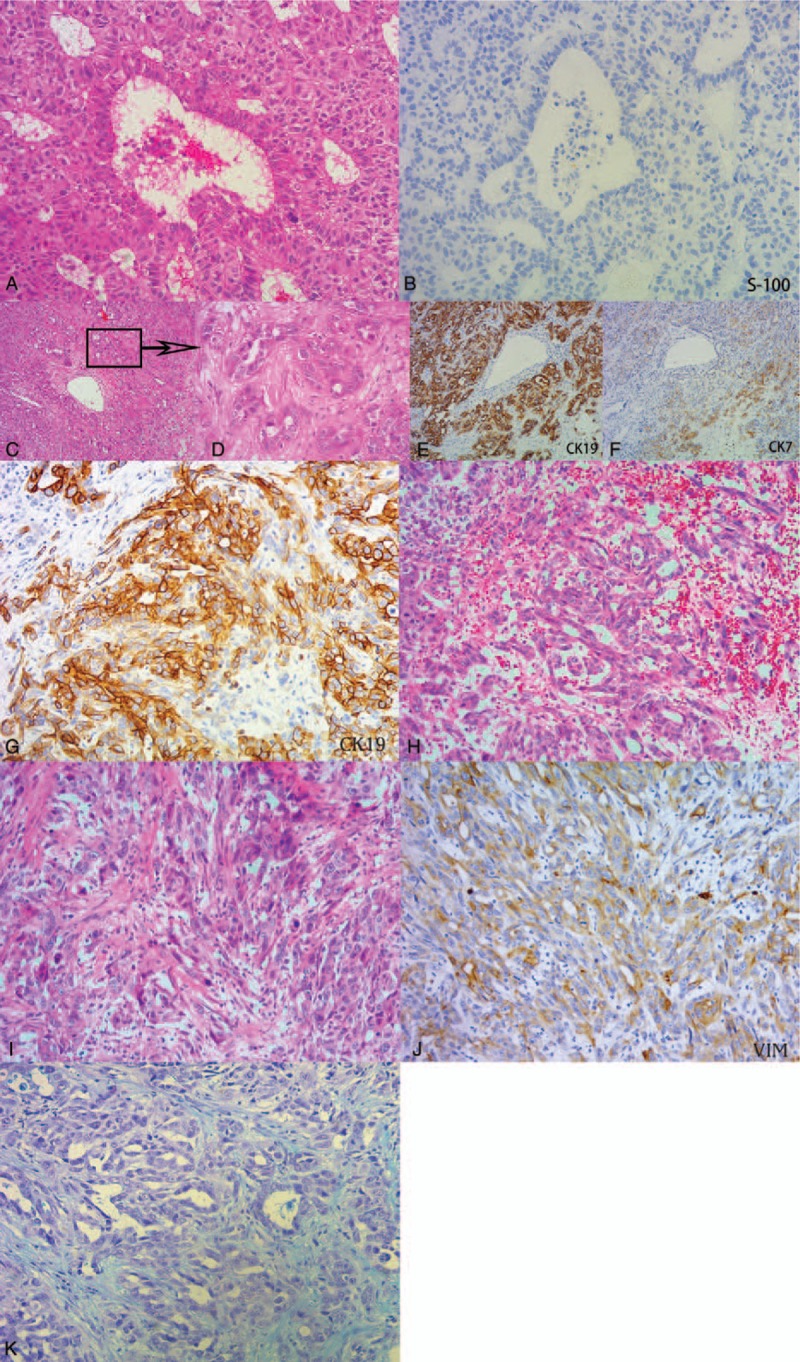
(A–B) The first case, HCC component. (A) Typical, moderately differentiated hepatocellular carcinoma, trabeculae were variable thick, the tumor shows round, oval, inconsistent cells. (HE, 200X×). (B) Tumor cells were negative for S-100. (IHC, 200×). (C–F) The first case, CC component. (C–D) Cholangiocarcinoma component, composed of distorted tubular glands with an abundant fibrous stroma, tumor cells had vesicular nuclei, obvious nucleoli and slightly eosinophilic cytoplasm. (HE, 100×, 400×). (E–F) Tumor cells were positive for CK19, CK7. (IHC, 100×). (G–H, K) The first case, mixed area. (G–H) Sarcomatoid component mixed with cholangiocarcinoma, cholangiocarcinoma cells, and spindle cells were positive for CK19 (IHC, 200×; HE, 200×). (K) CC cells and some spindle cells secrete acid mucopolysaccharides (blue). (AB-PAS, 200×). (I—J) The first case, sarcomatoid component. (I) The sarcomatoid component was composed of short shuttle-like cells with clear nucleoli and few mitotic figures (HE, 200×). (J) Tumor cells were positive for VIM. (IHC, 200×).

The resected specimen measured 2.5 × 2.5 × 2.0 cm. The tumor was not capsulated, but its margin was well circumscribed. Microscopic examination showed a heterogeneous pattern. Compact trabeculae were variable cells thick as observed in HCC. Therefore, immunohistochemical stains for neuroendocrine markers, nuclear cell adhesion molecule (CD56) and S-100, were used to exclude neuroendocrine differentiation. Both of them were negative. The tumor showed round, oval, inconsistent cells (Fig. 1A–B). The stroma was composed of sinusoid-like blood spaces lined by a single layer of endothelial cells that were different from those in normal liver tissue. They lacked normal capillaries, including immunohistochemically demonstrable, CD34 staining. Areas of CC were composed of moderately distorted, tubular glands with cribriform formations and cord-like patterns. Tumor cells had nucleoli and slightly eosinophilic cytoplasm (Fig. 1C–F). The sarcomatoid component was composed of spindle-shaped cells. Mitotic figures were present (Fig. 1I–J). In addition, some CC intermingled with spindle-shaped cells (Fig. 1G–H, K). Atypical mitoses and necrosis were present throughout the specimen. The relative proportions of the tumor consisted of 15% of HCC, 35% of CC, 25% of CC mixed with spindle-shaped cells, and 25% of sarcomatoid component.

Immunohistochemical stains revealed that the moderately differentiated HCC component showed focal strongly positive staining for Heppar1 and Glypican-3 (GPC-3), and negative staining for AFP, cytokeratin (CK) 7, CK19, CD56, and S-100. The CC component was positive for CK7, CK19, Mucin-core protein 1 (Muc-1),^[9]^ and epithelial adhesion molecule (EpCAM/Moc-31),^[10]^ and negative for Heppar1, GPC-3, and AFP. Spindle cells were negative for Heppar1. CD117 (c-KIT), CD56, and CD133 were negative in both sarcomatoid and carcinoma components (Table 1).

**Table 1:**
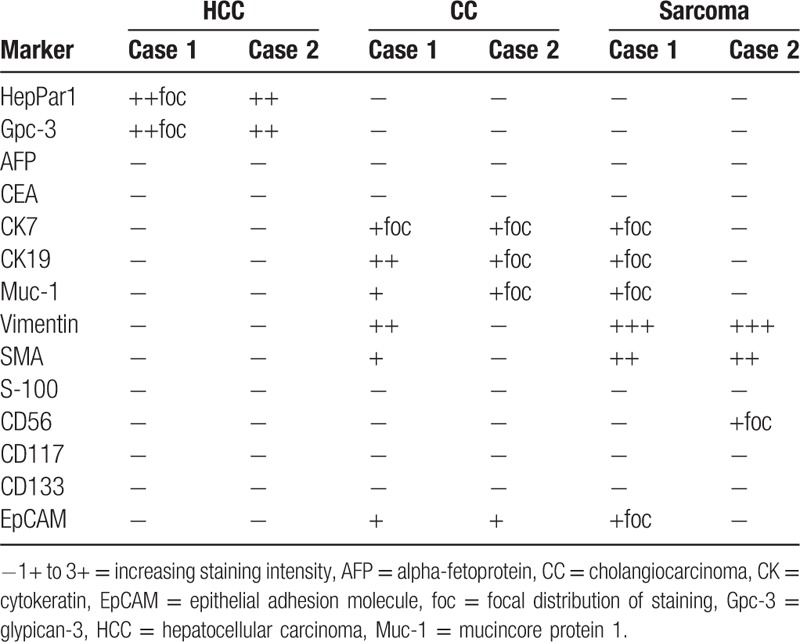
Phenotypic, immunohistochemical expression in the 3 morphologically different tumor.

### 2.2 Case 2

A 54-year-old man was admitted to our hospital for abnormal stools. He had a past history of hepatitis B-related cirrhosis. Imaging studies showed a 4 × 3 cm mass in the middle of the falciform ligament. Full blood cell count showed a declining red blood cell count of 3.32 × 10^12^/L (4.30–5.80 × 10^12^/L), hemoglobin 9.8 g/L (130–175 g/L). Serum liver function test results were normal. Serum AFP level was at 17.85 ng/mL (0–9 ng/mL). Hepatitis B surface antigen, E antigen, and C antibody were positive; hepatitis B surface antibody and hepatitis C antibody were negative. The patient subsequently underwent laparoscopic, hepatic left lateral lobectomy. At postoperative day 30, his serum AFP level was normal (7.95 ng/mL). The patient had no evidence of recurrence or metastasis during the 8 months of follow-up.

**Figure 2:**
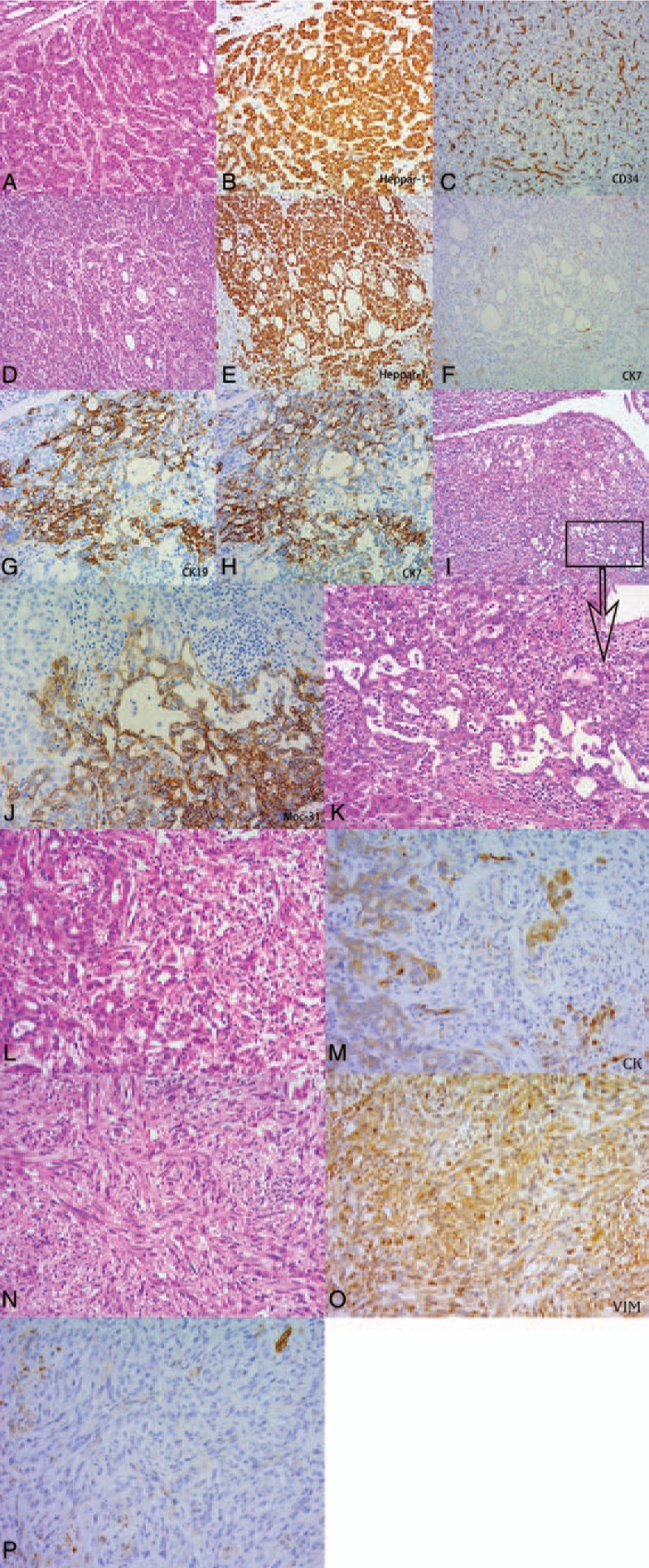
(A–C) The second case, HCC component. (A) Compact trabecula was 2 to 3 thickness (HE, 200×). (B–C) tumor cells were positive for Heppar-1 and sinusoid-like blood spaces stained positive for CD34. (IHC, 200×, 100×). (D–F) The second case, pseudoglandular component. (D) Pseudoglandular admixed with the trabecular pattern, the structure is formed by a single layer of cuboidal tumor cells. (HE, 100×). (E–F) Tumor cells were positive for Heppar-1 and negative for CK7. (IHC,100×). (G–K) The second case, CC component. (I, K) Cholangiocarcinoma component consisted of a single layer of tumor cells with vesicular nuclei, obvious nucleoli (HE, 100×, 200×). (G–H, J) Tumor cells were positive for CK19, CK7, and Moc-31 (IHC, 200×). (L–M) The second case, transitional forms. (L) The tumor cells change their morphology from atypia, bizarre shape to typical spindle shape. (HE, 200×). (M) Transition zone cells were positive for CK (IHC, 200X). (N–P) The second case, sarcomatoid component. (N) Extensive spindling of neoplastic cells is obvious, whirls and bundles are irregularly arranged and contain elongated cells (HE, 200×). (O–P) Tumor cells were positive for VIM and negative for CK (IHC, 200×).

The surgical resection specimen (3.5 × 3 × 3 cm) showed a well-demarcated tumor embedded in cirrhotic liver parenchyma with an intact tumor capsule. Microscopically, HCC with variable characteristic was observed. Tumor cells with atypia and increased nucleus-to-cytoplasm ratio showed slightly vacuolated nuclei and clear nucleoli, and were seen separated by sinusoid-like blood spaces. The tumor cells were positive staining for Heppar1, and negative staining for CK7, CK19, and Muc-1 (Fig. 2A–C). The CC component consisted of a single layer of tumor cells with vesicular nuclei, obvious nucleoli that expressed CK7, CK19, Muc-1, and EpCAM (Fig. 2G–K). Possible transitional forms were present, the intimate coexistence of both carcinoma and sarcomatous cells with a transitional form as well as positive staining for keratin (CK) in sarcomatous cells (Fig. 2L–M). Spindling of the neoplastic cells with bundles and whirls were observed, as typically found in sarcomas (Fig. 2N). A typical mitoses and necrosis were found throughout the specimen. The relative proportions of the tumor consisted of 45% of HCC, 20% of CC, 5% of transitional area, and 30% of sarcomatoid component.

Immunohistochemical studies showed that the HCC component stained positive for Heppar1 and GPC-3, and negative for AFP and carcinoembryonic antigen (CEA). Tumor with pseudoglandular pattern was positive for Heppar-1, GPC-3, but negative for CK7, CK19, and MUC-1 (Fig. 2D–F). The CC component stained positive for CK7, CK19, Muc-1, and Moc-31, and negative for Heppar1, GPC-3, and AFP. The sarcomatoid component stained strongly positive for vimentin (VIM) and SMA, partially positive for CD56, and negative for S-100, Heppar1, GPC-3, CK, CK7, CK19, and MUC-1. The transition zone cells stained positive for CK, CK7, and VIM. The sarcomatoid component was positive for VIM and SMA, and negative for CK, CK7, and CK19 (Table 1).

## 3 Discussion

According to the WHO classification (2010), cHCC-CC may be categorized into “classical”-type cHCC-CC and subtypes with stem cell features.^[11]^ The classical type contains areas of hepatocellular carcinoma (HCC), CC, and transitional zones. HCC from case 1 shows a moderately differentiated tumor, and from case 2, a well-differentiated tumor.

Diagnoses of both cases are not difficult. Immunohistochemical staining for Heppar1 is positive in >90% of HCC.^[12]^ GPC-3 is also expressed in HCC.^[13]^ In both cases the HCC component stains positive for Heppar1 and GPC-3, and negative for AFP. The CC component usually has a tubular growth pattern of variable-sized lumina and acinar, being cord-like, and with micropapillary features.^[14]^ In case 1, the CC component shows moderately distorted tubular glands with cribriform and cord-like patterns, accompanied by abundant stroma. In case 2, there is a typical well-differentiated adenocarcinoma. In both cases, the CC components stain positive for MUC-1, CK7, CK19, and EpCAM. Morphologically, the 2 cell types (HCC cells and CC cells) can exist in 1 of 3 forms: separately, contiguously but independently, or intermingled within a mass.^[7]^ According to a study of cHCC-CC, Wakasa et al consider that the CC component originates from HCC.^[15]^ In our study, we observe that the HCC component is independent of the CC component in case 1, but intermixed with CC cells in case 2. Whether the CC component derives from a dedifferentiation of ordinary HCC need further investigation.

Sarcomatoid changes are rarely seen in epithelial malignancy. Morphology varies from spindle shape, to pleomorphic of round and oval and epithelioid cells. Mitotic rates are usually high and atypical mitoses are frequent.^[16]^Immunohistochemically, the sarcomatous cells of CC component in case 1 are strongly positive for mesenchymal markers, such as VIM and SMA, and focally positive for epithelioid markers such as CK7 and CK19. They are negative for Heppar1 and GPC-3. Partial epithelial (CC component) and sarcomatous areas are tightly intermingled. Poorly differentiated adenocarcinoma has round, spindle cells with low adhesiveness; under the circumstances, sarcomatous change and poorly differentiated adenocarcinoma are difficult to distinguish. But CC cells have neutral or acidic mucosubstance in cytoplasm. We can identify the mucosubstance through AB-PAS staining.^[17]^ The mixed area of case 1, the AB staining, was partial positive in spindle cells (Fig. 1L). It suggests that partial spindle-shaped cells are poorly differentiated adenocarcinoma, and other spindle cells may derive from a dedifferentiation of ordinary CC. In case 2, epithelial and mesenchymal components express both epithelial markers and mesenchymal markers. The sarcomatoid component is diffusely positive for VIM and SMA, partially positive for CD56, and negative for Heppar1, GPC-3, CK7, CK19, MUC-1, and S-100. In the transition area, the tumor cells change their morphology from atypia, bizarre shape to typical spindle shape.

Clinically, sarcomatoid components of these tumors may have a high metastatic potential to the portal vein and central venous system.^[1–8]^ However, in case 1 the patient has survived for 10 years without any postoperative treatment. The patient is 44-year-old with good physique and the size of lesion is small. The prognosis may be related with the body quality and tumor size. The second patient (case 2) is free of disease at 8 months of postoperative follow-up. To measure this, extended follow-up may be necessary. Prognosis for combined HCC/CC with sarcomatoid features faces conflicting evidence from very few cases. Long-term follow-ups of more patients are needed.

A summary of the 8 cases is shown in Table 2. The tumors most often occur in men; the mean age of the patients is 61 years old. Serum marker for hepatitis C virus antibody is positive in 1 case, and HbsAg is positive in 3 cases. Two cases undergo transarterial embolization therapies, and 2 cases undergo transarterial chemoembolization. Four of 8 cases have metastasis,^[1,3,4,7]^ and 6 patients die from this disease.^[1–4,7,8]^Most reported cases of primary hepatic lesion are diffusely involved in necrosis.^[1,3,6–8]^ On microscopic examination, the epithelial components of all 8 tumors are HCC–CC with varied differentiation. The sarcomatoid area is composed of atypical spindle-shaped, epithelioid or small polygonal cells, whereas osteoid cells are found in 1 case.^[1]^ The sarcomatoid area is observed in both HCC and CC components in 4 cases,^[1,2,4,8]^ mainly in the CC component in 3 cases,^[3,5,7]^ and in the HCC component in only 1 case.^[6]^ In conclusion, HCC–CC with sarcomatous change is an extremely rare primary hepatic malignancy, of which only a few cases have been reported. Further investigations are needed to fully identify the clinicopathological features.

**Table 2:**
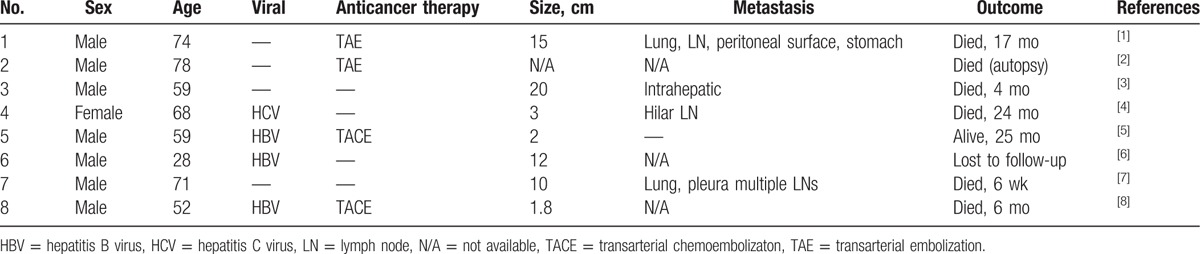
Previously reported cases of combined hepatocellular-cholangiocarcinoma with sarcomatous change.
